# The model homologue of the partially defective human 5,10-methylenetetrahydrofolate reductase, considered as a risk factor for stroke due to increased homocysteine level, can be protected and reactivated by heat shock proteins

**DOI:** 10.1007/s11011-016-9844-8

**Published:** 2016-05-28

**Authors:** Michał Grabowski, Bogdan Banecki, Leszek Kadziński, Joanna Jakóbkiewicz-Banecka, Magdalena Gabig-Cimińska, Alicja Węgrzyn, Grzegorz Węgrzyn, Zyta Banecka-Majkutewicz

**Affiliations:** 1 Intercollegiate Faculty of Biotechnology, University of Gdańsk and Medical University of Gdańsk, Kładki 24, 80-822 Gdańsk, Poland; 2 Department of Molecular Biology, University of Gdańsk, Wita Stwosza 59, 80-308 Gdańsk, Poland; 3 Laboratory of Molecular Biology (affiliated with the University of Gdańsk), Institute of Biochemistry and Biophysics, Polish Academy of Sciences, Wita Stwosza 59, 80-308 Gdańsk, Poland; 4 Department of Neurology, Medical University of Gdańsk, Dębinki 7, 80-211 Gdańsk, Poland

**Keywords:** 5,10-methylenetetrahydrofolate reductase, *Escherichia coli* MetF protein, Human *MTHFR* gene, Homocysteine, Heat shock proteins

## Abstract

The A222 V substitution in the human *MTHFR* gene product (5,10-methylenetetrahydrofolate reductase) is responsible for a decreased activity of this enzyme. This may cause an increased homocysteine level, considered as a risk factor for arteriosclerosis and stroke. The bacterial homologue of the human enzyme, MetF, has been found to be a useful model in genetic and biochemical studies. The similarity of *Escherichia coli* MetF and human MTHFR proteins is so high that particular mutations in the corresponding human gene can be reflected by the bacterial mutants. For example, the A222 V substitution in MTHFR (caused by the C667T substitution in the *MTHFR* gene) can be ascribed to the A117 V substitution in MetF. Here, it is reported that a temperature-sensitive MetF117 (A117 V) protein can be partially protected from a thermal inactivation by the heat shock proteins from the Hsp70/100 systems. Moreover, activity of the thermally denatured enzyme can be partially restored by the same heat shock proteins. High temperature protein G (HtpG) had no effect on MetF117 activity in both experimental systems. The presented results indicate that functions of heat shock proteins may be required for maintenance of the MetF117 function. This may have implications for the mechanisms of arteriosclerosis and stroke, especially in the light of previous findings that the A222 V MTHFR polymorphism may be a risk factor for stroke, as well as recently published results which demonstrated the increased levels of antibodies against heat shock proteins in stroke patients.

## Introduction

**Fig. 1:**
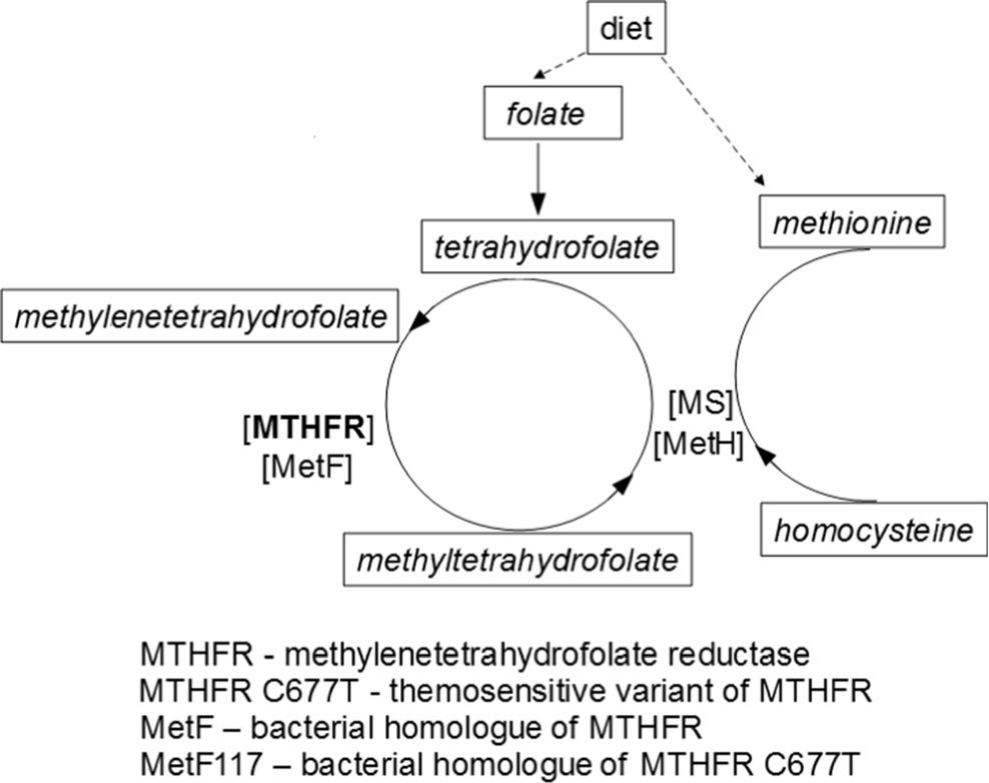
Homocysteine metabolic pathway diagram detailing the human and bacterial methylenetetrahydrofolate reductase. MTHFR – human methylenetetrahydrofolate reductase, MetF – *E.coli* methylenetetrahydrofolate reductase, MS – methionine synthase, MetH – *E.coli* methionine synthase

5,10-Methylenetetrahydrofolate reductase (EC 1.5.1.20) is encoded in humans by the *MTHFR* gene. This enzyme is involved in homocysteine metabolism (presented schematically in Fig. 1), as it catalyses the conversion of 5,10-methylenetetrahydrofolate to 5-methyltetrahydrofolate, which serves as a methyl donor in the remethylation of homocysteine to methionine (Bailey and Gregory 1999). Therefore, dysfunction or decreased activity of the *MTHFR* gene product leads to increased levels of homocysteine. Hyperhomocysteinemia causes toxicity to vascular and nervous systems (Perła-Kaján et al. 2007). Thus, this metabolic defect is considered a risk factor for various diseases, including stroke, minimal cognitive impairment, dementia, Parkinson disease, multiple sclerosis, epilepsy, and eclampsia (for reviews see Ansari et al. 2014; Keshteli et al. 2015).

Among diseases for which hyperhomocysteinemia is a risk factor, stroke is one of the most severe disorders (for a review, see Banecka-Majkutewicz et al. 2012). Increased levels of homocysteine was correlated with the incidence of stroke in as various populations as those from Poland (Sawuła et al. 2009) and Karachi (Sadiq et al. 2014). Recent studies indicate that in stroke patients there are increased levels of antibodies against heat shock proteins (Hsps) (Banecka-Majkutewicz et al. 2014). Such results might suggest an essential role for Hsp deficiency in development of the atherosclerosis and stroke. However, the exact role of Hsps in putative stroke prevention remains unclear. Some light on this problem was shed by studies demonstrating that the Hsp70/100 system has a role in maintaining activity of *Escherichia coli* MetH protein, a homologue of human methionine synthase, another enzyme involved in homocysteine metabolism (Grabowski et al. 2012; Fig. 1). Since there is a high similarity between *E. coli* MetH and human methionine synthase, one might suggest that increased levels of anti-Hsps antibodies might reduce the efficiency of the chaperone systems, thus interfering with the homocysteine metabolism.

Continuing this line of studies, we aimed to investigate a potential role for Hsps in modulation of activity of the *E. coli* MetF protein, a homologue of the human 5,10-methylenetetrahydrofolate reductase (MTHFR). The level of similarity between these enzymes is so high that particular amino acid residues in MetF can be ascribed to their counterparts in MTHFR. For example A117 in MetF corresponds to A222 in MTHFR (Guenther et al. 1999). On the basis of structural and biochemical properties of the mutated form of MetF (A117 V), a specific mechanism leading to decreased activity and increased thermolability of the A222 V variant of MTHFR has been suggested (Guenther et al. 1999). The A222 V substitution is an effect of a common polymorphism in the *MTHFR* gene, the C to T transition at nucleotide position 677 (Ueland et al. 2001). It was demonstrated that human homozygotes with two 677 T alleles have a high concentration of plasma homocysteine, while heterozygotes may reveal moderate hyperhomocysteinemia, relative to 667C homozygotes (Kluijtmans and Whitehead 2001). Subsequent studies indicated that the *E. coli* system, in which production of wild-type or the A117 V variant of the MetF enzyme or both is allowed, can efficiently mimic human wild-type homozygote, A222 V homozygote or heterozygote, respectively, in activities of methylenetetrahydrofolate reductase (Jakobkiewicz-Banecka et al. 2005). This indicated that the *E. coli* MetF protein can be successfully employed as a model in studies on the human MTHFR enzyme, and has opened new possibilities of more detailed investigations, including aspects related to human diseases. In fact, there is no doubt that experiments with bacterial systems are significantly simpler and easier to work with than studies on human subjects, thus, the bacterial model of the human enzyme is particularly useful. It was demonstrated that among other phenotypes, bacteria expressing the *metF117* allele produces a thermosensitive 117 V variant of MetF which biochemically and physiologically corresponds to a thermolabile A222 V variant of human MTHFR (Jakobkiewicz-Banecka et al. 2005). Therefore, in this work, we aimed to use the MetF117 protein to test whether Hsps can contribute to the function of the thermolabile methylenetetrahydrofolate reductase.

## Materials and methods

### Proteins

Overproduction of proteins was performed in *E. coli* BL21(DE3) RIL in TB medium with 5 M FAD. The wild-type MetF protein was purified as described previously (Sheppard et al. 1999). The 117 variant of MetF was purified analogously as the wild-type enzyme, but all buffers used for purification of MetF117 were supplemented with 5 μM FAD.

DnaK, GrpE, DnaJ, ClpB and HtpG were overproduced and purified according to the previously published methods (Banecki and Zylicz 1996; Banecki et al. 1996; Spence and Georgopoulos 1989; Woo et al. 1992; Wawrzynów et al. 1995).

### Determination of methylenetetrahydrofolate reductase activity

Determination of methylenetetrahydrofolate reductase was performed as described previously (Sheppard et al. 1999), by measuring a decrease in absorbance of NADH, consumed during the reaction. The reaction mixture consisted of 50 mM phosphate buffer containing 10 % glycerol, 0.3 mM EDTA, 400 μM NADH, and 1.4 mM menadione (vitamin K3 was used as an artificial substrate for MetF). The activity of MetF was determined by measurement of the kinetics of the reaction at 37 °C. The reaction mixture was prepared without the enzyme, and incubated for 5 min. Following reaction initiation by the addition of 0.3 μM enzyme, the measurement was carried out for 30 min, by monitoring the absorbance at a wavelength of 340 nm.

### Hsps protection of MetF117 from temperature-mediated denaturation

MetF (at final concentration of 0.3 μM) was pre-incubated in the presence of 50 mM phosphate buffer (pH 7.2) containing 10 % glycerol, 0.3 mM EDTA, 50 mM NaCl, 20 mM KCl, 20 mM MgCl_2_, and in the presence of DnaK, DnaJ, GrpE, ClpB (the Hsp 70/100 system) or HtpG, and 5 mM ATP. The concentrations of heat shock proteins were 3.7 μM, 1.4 μM, 0.36 μM, 1.5 μM, respectively for Hsp70/100, and 3.6 μM for HtpG. The incubation was carried out for 15 min at 50 °C. Finally, the samples were subjected to the MetF activity test.

### Reactivation of thermally-denatured MetF117 by Hsps

MetF (at final concentration of 0.3 μM) was thermally inactivated by 15 min incubation at 50 °C in the presence of 50 mM phosphate buffer (pH = 7.2) containing 10 % glycerol, 0.3 mM EDTA, 50 mM NaCl, 20 mM KCl, and 20 mM MgCl_2_. Following the incubation, the enzyme was renatured in the presence of Hsp 70/100 or HtpG at the concentrations indicated in the preceding subsection. The renaturation was carried out for 45 min at 20 °C. Finally, the samples were subjected to the MetF activity test.

### Statistical analysis

Statistical analysis was performed using T-test. A *p* value < 0.05 was considered to indicate statistical significance. Each experiment was repeated three times. All data were calculated with Statistica 12 software (StatSoft).

## Results

**Fig. 2:**
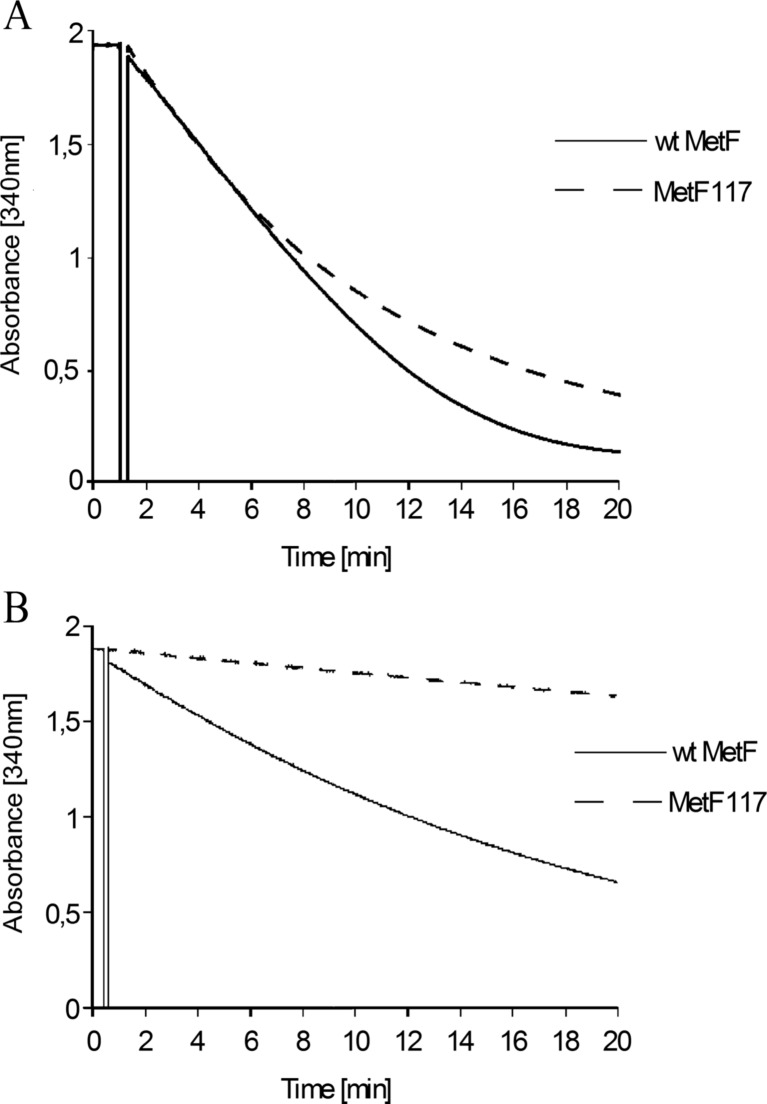
The reaction kinetics of wild-type MetF and MetF117 at 37 °C (panel **a**) and at 50 °C (panel **b**). The enzymatic activity is reflected by a decrease in NADH absorbance, as this compound is consumed during the reaction

Wild-type and the 117 V variant of *E. coli* methylenetetrahydrofolate reductase, MetF and MetF117, respectively, were purified from bacteria overexpressing corresponding *metF* alleles from a plasmid. We found that both variants of the enzyme had similar activities at 37 °C (Fig. 2a).

To test effects of an increased temperature on MetF activity, the reaction mixtures were incubated at various temperatures. Significantly higher thermosensitivity of MetF117, relative to the wild-type enzyme, was observed (Fig. 2b). For further experiments, temperature 50 °C was chosen, at which the difference between both variants of the enzyme was the most pronounced.

**Fig. 3:**
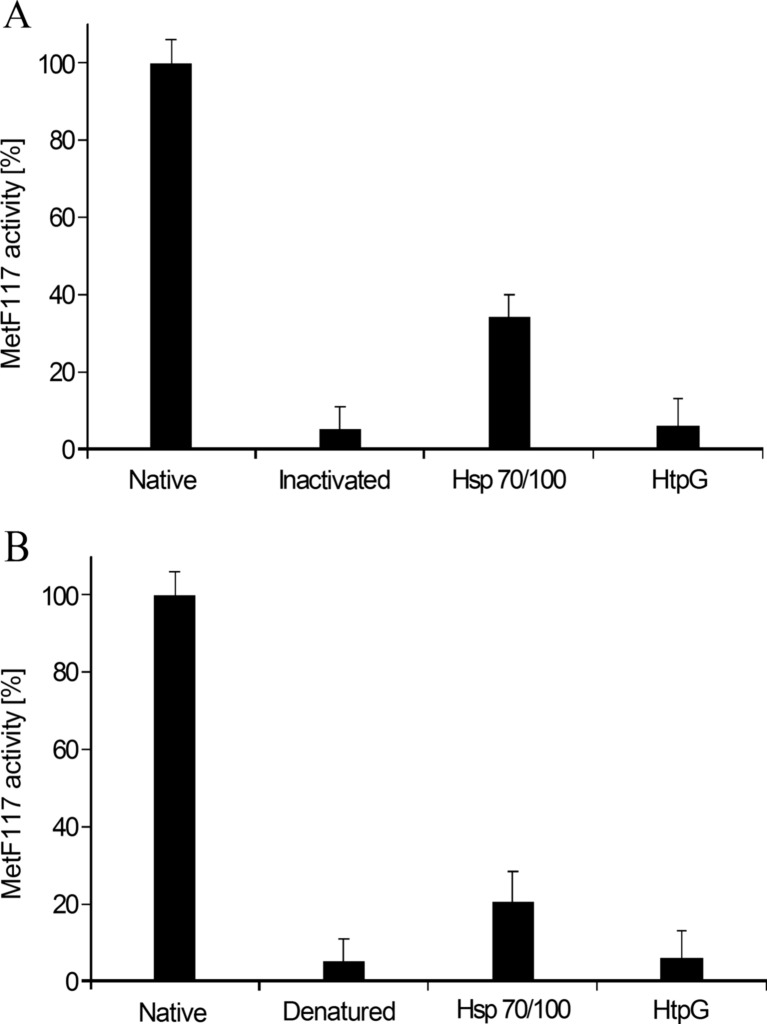
The protection against heat-mediated inactivation (panel **a**) and reactivation of thermally-denatured (panel B) MetF117 enzyme by Hsp70/100 and HtpG hsps. Control experiments (Native) were performed at 37 °C. Heat-mediated inactivation reaction (panel **a**) was carried out for 15 min at 50 °C in the absence (Inactivated) or presence of Hsp70/100 or HtpG. Denaturation of the enzyme (panel **b**) was performed by its incubation for 15 min at 50 °C, and the reactivation reaction was carried out for 45 min at 20 °C in the absence (Denatured) or presence of Hsp70/100 or HtpG

We found that the proteins from the Hsp70/100 system, DnaK, DnaJ, GroEL, ClpB, could partially protect the MetF117 protein against thermal denaturation (Fig. 3a). The mutated enzyme revealed a significantly higher activity at 50 °C in the presence of the Hsp70/100 systems than in the absence of Hsps (*p* = 0.0012; t-test). HtpG had no significant effect on MetF117 activity (*p* = 0.4667; t-test).

When testing a reactivation of the previously thermally-denatured MetF117, we found that the Hsp70/100 system is able to partially recover the enzyme activity (Fig. 3b; *p* = 0.015; t-test). Again, HtpG was unable to restore MetF117 activity (*p* = 0.5; t-test). Therefore, we conclude that Hsps can both partially protect and rescue MetF117 activity at elevated temperature.

## Discussion

The results presented in this report indicate that Hsps have important roles in activation of the mutated MetF variant. The in vitro experiments were performed at 50 °C to demonstrate the most pronounced effects which occurred at an extremely high temperature relative to physiological conditions. However, most probably, similar functions of the Hsp70/100 system may be important at physiological temperatures in living organisms. This assumption is based on the fact that at higher temperature the negative effects on the protein function should be more pronounced than at a lower one, thus, their correction is more difficult. Moreover, the Hsp70/100 system should work more efficiently under physiological conditions than at the extremely high temperature. Hence, if the system works under drastic conditions, it is likely that it also works in the living organism. Unfortunately, in the artificial in vitro system, the easily detected and unequivocally measurable effects occurred only at high temperature. On the other hand, this system provided a possibility to obtain a clear response to the asked question, due to its simplicity and the presence of only those factors which activities were investigated.

Because of the high similarity, both structural (Guenther et al. 1999) and functional (Jakobkiewicz-Banecka et al. 2005), between bacterial MetF and human MTHFR proteins, and their thermosensitive variants 117 V and 222 V, respectively, we suggest that some implications can be proposed for the human organism. The 667 T allele of the *MTHFR* gene has been shown to cause a decreased activity of the encoded enzyme, and an increased levels of homocysteine, even if occurring in the heterozygotic state (Kluijtmans and Whitehead 2001). Moreover, hyperhomocysteinemia was considered a risk factor for stroke (Sawuła et al. 2009; Ansari et al. 2014; Sadiq et al. 2014). Nevertheless, it is obvious that not all persons having an increased plasma level of homocysteine suffer from stroke. Therefore, other factors, agents and/or conditions must also be involved in the mechanism of an increased risk of this disorder in persons with hyperhomocysteinemia. One of them may be Hsps, as these proteins can modulate activity of methylenetetrahydrofolate reductase, and particularly its thermolabille form.

It is tempting to speculate that if the Hsp70/100 system is fully functional, the activity of the thermolabille MTHFR is sufficient to keep the homocysteine level low enough to avoid an increased risk for stroke. However, it was reported that in stroke patients, as well as patients in whom stroke occurred in the past, there are increased levels of anti-Hsps antibodies (Banecka-Majkutewicz et al. 2014). These antibodies could inactivate Hsps, resulting in less efficient protection or reactivation of the mutant MTHFR, and in turn, increased levels of homocysteine, leading to a high risk for stroke. Along to this hypothesis, it is worth reminding that the Hsp70/100 system can also maintain appropriate activity of methionine synthase (Grabowski et al. 2012), and dysfunction of this enzyme can also cause hyperhomocysteinemia (Matthews and Elmore 2007). Intriguingly, HtpG had no effect on protection or renaturation of MetF117, thus, these reactions appear to be specific to the Hsp70/100 system. At the current stage of our knowledge, the reason and mechanism of such specificity are unclear and require further extensive studies, which extend beyond the aims of this study. We are aware that such a proposal requires experimental and clinical verifications in studies on the human biological material, nevertheless, this report indicates a possibility by which different factors might interplay in the way of stroke occurrence.
